# Review on Polymer-Based Composite Electrolytes for Lithium Batteries

**DOI:** 10.3389/fchem.2019.00522

**Published:** 2019-08-08

**Authors:** Penghui Yao, Haobin Yu, Zhiyu Ding, Yanchen Liu, Juan Lu, Marino Lavorgna, Junwei Wu, Xingjun Liu

**Affiliations:** ^1^Shenzhen Key Laboratory of Advanced Materials, Department of Materials Science and Engineering, Harbin Institute of Technology, Shenzhen, China; ^2^Institute of Polymers, Composite, and Biomaterials, National Research Council, Portici, Italy

**Keywords:** polymer solid electrolytes, polymer, lithium-ion batteries, Li-ion conductivity, composite

## Abstract

Lithium-ion batteries have dominated the high performance and mobile market for last decade. Despite their dominance in many areas, the development of current commercial lithium-ion batteries is experiencing bottlenecks, limited by safety risks such as: leakage, burning, and even explosions due to the low-boiling point organic liquid electrolytes. Solid electrolyte is a promising option to solve or mitigate those issues. Among all solid electrolytes, polymer based solid electrolytes have the advantages of low flammability, good flexibility, excellent thermal stability, and high safety. Numerous researchers have focused on implementing solid polymer based Li-ion batteries with high performance. Nevertheless, low Li-ion conductivity and poor mechanical properties are still the main challenges in its commercial development. In order to tackle the issues and improve the overall performance, composites with external particles are widely investigated to form a polymer-based composite electrolyte. In light of their work, this review discusses the progress of polymer-based composite lithium ion's solid electrolytes. In particular, the structures, ionic conductivities, electrochemical/chemical stabilities, and fabrications of solid polymer electrolytes are introduced in the text and summarized at the end. On the basis of previous work, the perspectives of solid polymer electrolytes are provided especially toward the future of lithium ion batteries.

## Introduction

From the moment in 1991 when the SONY corporation launched the commercialization of lithium-ion batteries, lithium-ion batteries have thrived significantly and dominated in many different applications, such as electric vehicles, portable devices (Scrosati and Garche, 2010; Verma et al., 2010; Manthiram et al., 2017). Although lithium-ion batteries have many advantages such as high energy density and long cycle life, the potential safety issues and saturated high energy density have become bottlenecks which impedes further development.

Current commercial lithium-ion batteries use liquid organic electrolytes, which have significant advantages of high conductivity and excellent wettability on electrode surfaces. However, the obvious and inevitable drawbacks of liquid electrolytes are electrochemical instabilities and potential risks, plus low ion selectivity. Compared with liquid electrolytes, solid electrolytes have higher safety and thermal stability, since it can provide a physical barrier layer to separate positive and negative electrodes and prevent thermal runaway under high temperature or impact. In addition, solid electrolyte makes it possible to use a lithium metal anode, due to its effective suppression of Li dendrite formation. Despite the significant advantages, some weaknesses still remain to be improved, such as low ionic conductivity and insufficient interface contact. Plenty of research is being conducted to conquer the weakness and develop new generation of solid lithium batteries (Tang et al., 2007; Zhao et al., 2012; Liu et al., 2013; Zhang Q. Q. et al., 2017). To meet the commercial requirements, high ionic conductivity, favorable mechanical properties, and outstanding interfacial stability with the electrodes are the most fundamental requirements for solid electrolytes (Fergus, 2010).

Inorganic solid electrolyte (ISE), solid polymer electrolyte (SPE), and composite electrolyte (CSE) are widely studied in lithium-ion batteries. Oxide group and sulfide group are two types widely used in ISE. Some of them [such as sulfide-based Li_10_GeP_2_S_12_ (Kamaya et al., 2011)] exhibit high conductivity equivalent to that of organic liquid electrolytes, but the issues of high processing difficulty, high cost, and large interface impedance restricts its wide application (Knauth, 2009; Fergus, 2010). SPEs not only have excellent electrochemical performance and high safety, but are also good in flexibility and process ability, which has high possibilities for use in next-generation high-energy batteries (Dias et al., 2000; Stephan and Nahm, 2006; Yarmolenko et al., 2018). In the meantime, it avoids the danger of Li metal dendrite growth (Meyer, 1998; Agrawal and Pandey, 2008; Tikekar et al., 2016). SPEs, including polyethylene oxide (PEO) (Farrington and Briant, 1979; Watanabe et al., 1999; Siqueira and Ribeiro, 2006), polycarbonate (Forsyth et al., 1997; Sun et al., 2014; Liu et al., 2015), and polysiloxane (Sun et al., 1996; Fonseca and Neves, 2002) have been extensively investigated. However, the ionic conductivity and mechanical strength of SPEs are still not ideal, which is the major obstacle to hamper their wide applications (Chen et al., 2016).

Different methods are adopted for improving the polymer electrolyte system. Typically, they can be categorized into two approaches: (1) Polymer/polymer coordination and (2) Composite polymer electrolyte.

**Figure 1:**
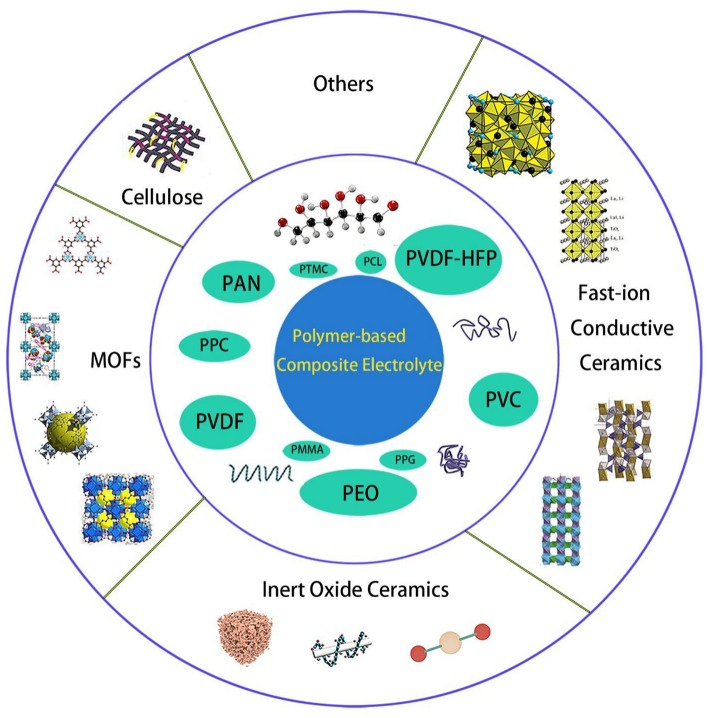
Categories of the existing polymer-based composite solid electrolytes.

Copolymerization, crosslinking, interpenetration, and blending are widely used as polymer/polymer coordination; however, it does not significantly increase the mechanical properties of the electrolyte. Various composites had been mixed into polymers, as shown in Figure 1, including inert ceramic fillers (Agrawal and Pandey, 2008; Lin et al., 2016), fast-ion conductive ceramics (Aliahmad et al., 2016; Keller et al., 2017; Ling et al., 2018), lithium salts (Do et al., 1996), ionic liquid (Subianto et al., 2009), etc. With the synergistic effect of polymer and inorganic filler, the room temperature conductivity and mechanical strength of composite polymer electrolyte can be greatly improved, as well as the interface stability with the electrode. In my group, similar synergistic effects on composite electrolyte had been reported in inorganic fillers composite with Nafion membrane for direct methanol fuel cell applications (Cui et al., 2015, 2018), the corresponding mechanism is similar with the composite electrolyte with organic fillers. The used polymer matrices and properties for SPE are summarized in Table 1.

**Table 1:** Common polymer matrix.

**Polymer matrix**	**Molecular formula**	**Glass transition temperature *T*_*g*_/^**°**^C**	**Melting point *T*_*m*_/^**°**^C**
PEO	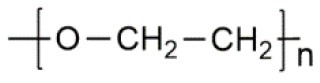	−64	65
PVC	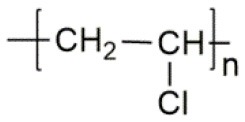	80	220
PAN	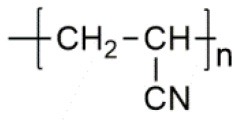	125	317
PMMA	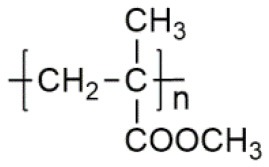	105	Amorphous
PVDF	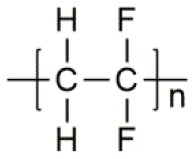	−40	171
PVDF-HFP	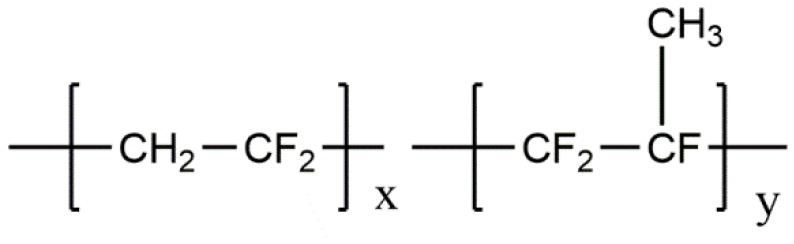	−90	135
PPG	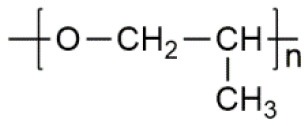	−60	Amorphous
PDMS	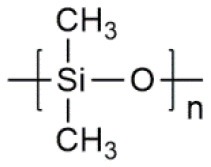	−127	−40
PEC	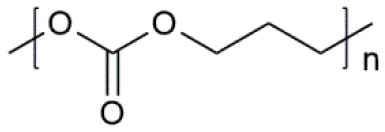	5	Amorphous
PPC	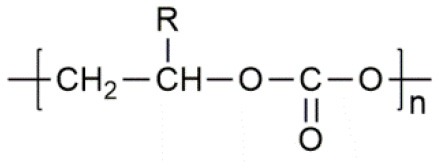	35	Amorphous
PCL	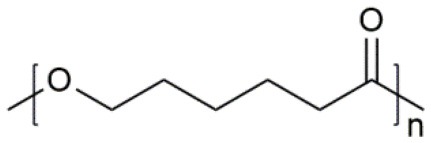	−60	Amorphous
PTMC	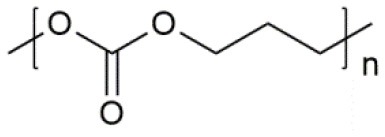	−15	Amorphous

**Figure 2:**
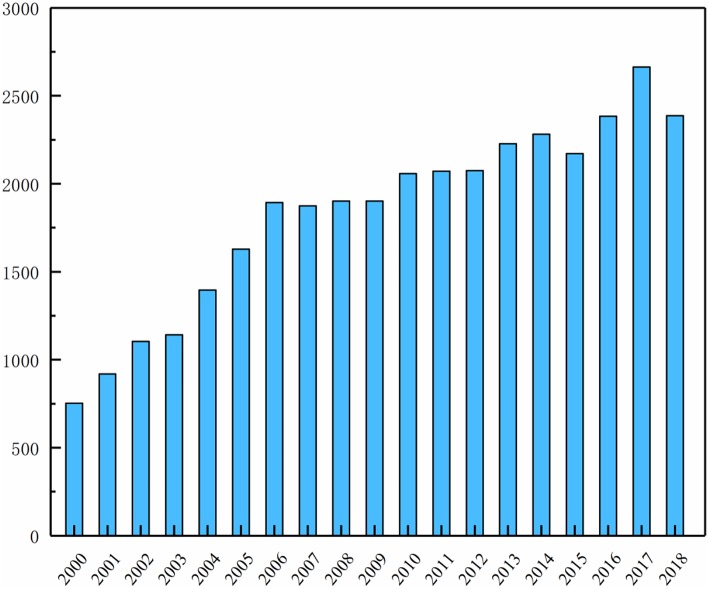
The number of published sci-hech articles in the polymer-based solid electrolyte over a period of 19 years from 2000 to 2019.

Solid polymer electrolytes (SPEs) currently have great application prospects in lithium batteries fabrication, numerous researchers also take great efforts to develop innovative SPEs and the successful applications will play a key role in developing lithium battery with excellent performance. Figure 2 shows that the number of published sci-tech articles in the polymer-based solid electrolyte over a period of 19 years from 2000 to 2018. The trend shows the steady increase from about 750 in 2000 to the largest point around 2,400 in 2017. From the year of 2010, the number of essays in this field keep steady over 2000, which is the fact that polymer-based solid electrolyte will have excellent application prospects. A large number of reviews have summarized the research and development history of polymer electrolytes (Qiu et al., 2004; Dong and Wang, 2005; Srivastava and Tiwari, 2009; Fergus, 2010; Liu et al., 2013; Osada et al., 2016; Zhang Q. Q. et al., 2017). However, there is relatively few reviews on polymer-based solid electrolytes.

This review article highlights recent researches on SPEs for solid state lithium-ion batteries, in particular about the effects of composition with various filler materials. In this review, polymer based composite electrolytes, including polymer/inert ceramics, polymer/fast-ion conductive, polymer/ionic liquid, polymer/MOFs, and polymer/cellulose composite electrolytes. Furthermore, a perspective on the future research direction for developing safety, stable, and high energy density composite polymer electrolytes for solid-state batteries will be provided.

## Ionic Conductivity and Interface

### Ionic Conductivity Mechanism

**Figure 3:**
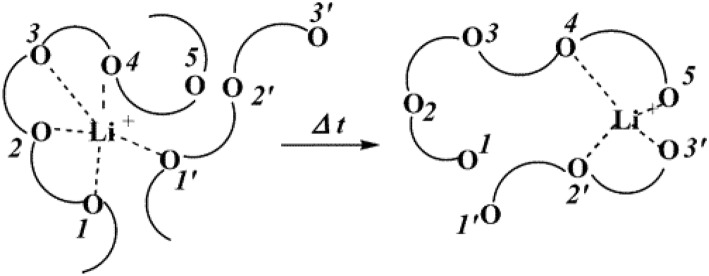
Schematic diagram of lithium ion conduction mechanism of PEO-based polymer electrolyte. [Reproduction with permission from Xu (2004), Copyright 2004, American Chemical Society].

In order to develop SPEs with high lithium ion conductivity, the polymer should not only dissolve lithium salt, but also be able to couple with lithium ions. The polar groups in the polymer (—O—, —S—, etc.) are effective building blocks for dissolving lithium salts. Most of the research on all-SPEs is focused on polyethylene oxide (PEO) and its derivatives. The lone pair of oxygens on the PEO segment is coordinated to the lithium ion by Coulombic interaction, causing the anion and cation of the lithium salt to dissociate. In the process, PEO acts as solvent, and the lithium salt dissolves into the PEO matrix. In addition to the oxygen atom (—O—) on the PEO chain, other atoms such as the nitrogen in the imide (—NH—) and the sulfur in the thiol (—S—) also play a similar role. Under the electric field, the migration movement of Li^+^ cations are from one coordination point to another along the polymer segment, or jump from one segment to another. The ion transport mechanism of polymer electrolytes such as PEO is shown in Figure 3 (Xu, 2004).

In the polymer-lithium salt composite system, the ions are not free to move due to the huge size of the polymer chain plus the boundary effect of crystalline domains. The factors affecting the ionic conductivity are the number of lithium ions and the mobility of the polymer chain. The amount of ions that can be migrated depends on the ability of the polymer to dissociate the lithium salt, and thus the lithium salt of low lattice energy and the polymer of high dielectric constant can promote this dissociation (Young et al., 2014). Under steady state conditions, the ionic conductivity can be expressed by the following equation (Wei-Min, 2012):

(1) $$\begin{array}{r} \sigma =\text{F }\sum n_{i}q_{i}\mu_{i} \end{array}$$

Here, F is the Faraday constant; *n*_*i*_ represents the number of free ions; *q*_*i*_ represents the number of charges, and μ_*i*_ is the mobility. Therefore, it can be seen that in the polymer electrolyte, the increasement of the concentration of the movable ions and the migration speed of the ions can increase the conductivity of the ions.

In SPEs, the most commonly used theory to explain the migration of ions in polymers includes Arrhenius theory, Vogel-Tammann-Fulcher (VTF) theory, William-Landel-Ferry (WLF) theory, and the combinations of above theories (Ratner et al., 2000; Quartarone and Mustarelli, 2011).

The classical Arrhenius theory explains the temperature relationship of ion migration caused by polymer segment motion, expressed as (Zhang Q. Q. et al., 2017):

(2) $$\begin{array}{r} \sigma =\sigma_{0}\exp\left(\frac{{-E}_{a}}{KT}\right) \end{array}$$

Here, *E*_*a*_ represents the activation energy for single molecules or groups of ions to migrate, σ_0_ represents the pre-exponential factor, while T represents thermodynamic temperature.

Generally, ion jump motion and polymer chain relaxation and/or segmental motion together affect conductivity, so the vs. 1/T curve is generally non-linear (Agrawal and Pandey, 2008). The typical lg-1/T in polymers is usually based on the *T*_*g*_-based equation, so VTF mainly describes the relationship between polymer electrolyte conductivity and temperature (Zhang Q. Q. et al., 2017):

(3) $$\begin{array}{r} \sigma =\sigma_{0}T^{-\frac{1}{2}}\exp\left(-\frac{B}{T-T_{0}}\right) \end{array}$$

Here, σ_0_ is a pre-exponential factor, B is an action factor with dimension as the energy dimension, and *T*_0_ is the reference temperature, which can be expressed in *T*_*g*_, normally 10–50 K below the experimental glass transition temperature. At room temperature, if only the effect of the polymer segment on conductivity is considered, low glass transition temperature can play a positive role in the improvement of the conductivity.

Based on the study of PEO and PPO salt complexes, the ionic conductivity can be related to frequency and temperature by using the William-Landel-Ferry (WLF) equation, considering the relaxation process of polymer molecular chain motion in an amorphous system. The expression is:

(4) $$\begin{array}{r} {\text{l}}_{\text{g}}\frac{\sigma \left(\text{T}\right)}{\sigma \left({\text{T}}_{\text{g}}\right)}=\frac{C_{1}\left(T-T_{g}\right)}{C_{2}+\left(T-T_{g}\right)} \end{array}$$

Here, σ(*T*_*g*_) is the conductivity of the relevant ions at glass transition temperature *T*_*g*_, and C_1_ and C_2_ are the WLF parameters in the free volume equation of ion migration, respectively.

*T*_*g*_ is one of the most critical parameter of polymer electrolyte. The conductivity is very low as the temperature below *T*_*g*_, and it will be obviously improved above *T*_*g*_. Therefore, to reduce *T*_*g*_ is beneficial to the improvement of conductivity.

The above three theories well-explain the conductive mechanism of the PEO-based electrolyte. The amorphous phase of the polymer is mainly effective for the migration of ions. The theory can also be applied to other polymer electrolytes.

### Interface

In the solid lithium-metal battery, the cathode is typically LiFePO_4_ or LiCoO_2_. Metallic lithium is used as a negative electrode. The cathode/electrolyte interface requires a solid electrolyte with excellent flexibility to ensure low interface resistance, while the anode/electrolyte interface requires a strong solid electrolyte to withstand the puncture of the metal lithium dendrites (Camacho-Forero and Balbuena, 2018; Wang L. P. et al., 2018; Zhang et al., 2018). The good flexibility of the SPE makes the lower interface resistance possible, but the low mechanical properties are difficult to withstand the puncture of the metal lithium dendrites. In contrast, a rigid inorganic ceramic electrolyte can withstand the metallic lithium dendrites, but has a large interfacial resistance due to insufficient contact with the electrodes (Xu et al., 2018). Therefore, the flexible polymer electrolyte or the rigid inorganic ceramic electrolyte has difficulty used in solid metal lithium battery separately. In order to take full advantage of polymer and inorganic ceramic electrolyte, polymer composite inorganic ceramic electrolyte offers an option. It is expected that the obtained solid metal lithium battery has both low interface resistance and the ability to inhibit lithium dendrite formation. In addition, the electrochemical instability of the interface easily leads to the occurrence of side reactions and thus the cover of the electrodes form a solid electrolyte interface (SEI), which may lead to a shortened cycle life of the cell (Xu et al., 2018).

## Solid Polymer Electrolytes With Inert Oxide Ceramics

In recent years, many studies have been addressed to incorporate inert oxide ceramics particles into polymer electrolyte, in order to improve the mechanical properties, reduce polymer crystallinity, and thus solve the problem of low ionic conductivity of SPE. Different types of inert ceramics had been incorporated into the polymer, such as SiO_2_ (Nan et al., 2003; Ketabi and Lian, 2013), Al_2_O_3_ (Weston and Steele, 1982; Capuano et al., 1991; Tambelli et al., 2002; Liang et al., 2015), TiO_2_ (Pal and Ghosh, 2018), zeolite, etc. The ionic conductivities of solid polymer composite electrolyte containing inert ceramic filler are showed in Table 2. In 1982, Weston and Steele (1982) mixed PEO with Al_2_O_3_ to form a composite. It was firstly proved that PEO doped with inert material particles exhibited an improvement of mechanical properties and ionic conductivity. Subsequently, Capuano et al. (1991) explored the contribution of the doping amount and particle size of LiAlO_2_ powder on the conductivity of solid electrolyte. It was found that the conductivity reached the highest as the doping amount of LiAlO_2_ was around 10 wt.%. It is also worth noting that particle size of the inert ceramic material affected the conductivity of the SPE, which increases with particle sizes as the size is <10 μm. Tambelli et al. (2002) reported that Al_2_O_3_ can effectively reduce the crystallinity and glass transition temperature of PEO. This confirms that the decrease of polymer crystallinity promotes the improvement of ionic conductivity. The decrease in crystallinity can enlarge the number of free segments of the polymer and accelerate the movement of the segments, which can effectively promote the migration of lithium ions. Similar results were reported on PEO-PMMA-LiTFSI-Al_2_O_3_ composite electrolytes. They were prepared based on PEO-PMMA as a host matrix and nano Al_2_O_3_ as filler by solution casting technique (Liang et al., 2015). The composite electrolytes doped with Al_2_O_3_ exhibited an improvement of the ionic conductivity from 6.71 × 10^−7^ to 9.39 × 10^−7^ S/cm.

**Table 2:** Summary of inert oxide Ceramics/polymer solid electrolytes.

**Year**	**Polymer electrolyte ingredients**	**Ionic conductivity (S/cm)**	**References**
1991	LiAlO_2_-PEO-LiClO_4_	10^−4^ (60°C)	Liu et al., 2013
1998	TiO_2_-PEO-LiClO_4_	10^−5^ (30°C)	Manthiram et al., 2017
2002	Al_2_O_3_-PEO-LiClO_4_	10^−2^ (60°C)	Liu et al., 2017
2003	SiO_2_-PEO-LiClO_4_-EC/PC	2 × 10^−4^ (25°C)	Lin et al., 2016
2015	Al_2_O_3_-PEO-PMMA-LiTFSI	9.39 × 10^−7^ (25°C)	Liu et al., 2016
2016	SiO_2_-Al_2_O_3_-PVDF-HFP-LiPF_3_(CF_3_CF_2_)_3_	10^−3^ (25°C)	Meyer, 1998
2016	Y_2_O_3_-doped ZrO_2_ nanowire (YSZ)—PAN–LiClO_4_	1.07 × 10^−5^ (30°C)	Mueller et al., 2006
2018	TiO_2−_PMMA-LiClO_4_	3 × 10^−4^ (30°C)	Liu et al., 2015
2018	Silica aerogel-PEO-LiTFSI	6 × 10^−4^ (30°C)	Liu et al., 2015
2018	Mg_2_B_2_O_5_ Nanowire-PEO-LiTFSI	1.53 × 10^−4^ (40°C)	Nair et al., 2009

SiO_2_ is also a common inert ceramic filler material used in the preparation of SPE. Lee et al. reported a composite of a PEO matrix and SiO_2_ fillers containing ethylene carbonate (EC)/propylene carbonate (PC). The composite had an ionic conductivity of 2 × 10^−4^ S/cm at ambient temperature (Nan et al., 2003) with 2.5 wt.% filler loadings. In addition to powder, SiO_2_ is also designed as a three-dimensional framework doped into the polymer. Lin et al. (2018) prepared a composite of PEO-Silica aerogel which exhibited high ionic conductivity 6 × 10^−4^ S/cm and high modulus 0.43 GPa. This study effectively solves the issue of poor mechanical properties and ionic conductivity of composite by controlling powder dispersion. SiO_2_ aerogel skeleton has a good acidic surface. It can interact with lithium cations extensively and form a continuous channel in the composite material, beneficial to salt dissociation and improvement of ionic conductivity. Ion pairs are difficult to form because of the strong Lewis acid-base interaction of doped TiO_2_ and the anion of lithium salt, resulting in more mobile charge carriers (Pal and Ghosh, 2018). Croce et al. (1998) studied a solid polymer electrode consisting of a nanosized TiO_2_ particles, PEO, and LiClO_4_. This hybrid exhibits a higher ionic conductivity of 10^−5^ S/cm. Pal and co-workers fabricated SPEs comprising of PMMA, LiClO_4_, and TiO_2_, by standard solution cast technique (Pal and Ghosh, 2018). The results showed that by composite nanosized-TiO_2_ to the polymer electrolytes, the thermal stability can be improved as well. The conductivity reached 3 × 10^−4^ S/cm at 303 K. Moreover, specific capacity of such polymer electrolyte-based LiCoO_2_/graphite at 30°C exhibited 30 mAh/g at room temperature in twelfth cycle. In addition, some studies have incorporated a variety of inorganic ceramics into the polymer, and the ionic conductivity has also been improved. For example, nanosized SiO_2_, and nanoporous Al_2_O_3_ were combined with PVDF-HFP to obtain composite electrolytes that delivered moderate conductivity of 10^−3^ S/cm with 2.5 wt.% of fillers (Aravindan and Vickraman, 2008).

**Figure 4:**
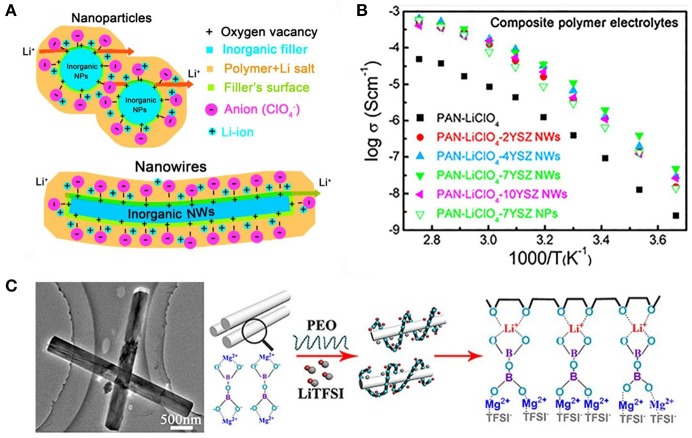
**(A)** Li-ion transport in the composite polymer electrolytes with Y_2_O_3_ nanoparticle and ZrO_2_ nanowire fillers. [Reproduction with permission from Liu et al. (2016) Copyright© 2016, American Chemical Society] **(B)** Relationship between the Y doping level and the conductivity, together with the conductivity of the YSZ bulk [Reproduction with permission from Liu et al. (2016) Copyright© 2016, American Chemical Society] **(C)** Schematics of lithium ion migration in Mg_2_B_2_O_5_ enhanced composite SSEs. [Reproduction with permission from Sheng et al. (2018) Copyright© 2018, American Chemical Society].

Liu et al. (2016) has the designed and fabricated a SPE comprising Y_2_O_3_ nanoparticle, ZrO_2_ nanowire fillers, and PAN by electrospinning (Figure 4A). Y_2_O_3_-stabilized ZrO_2_(YSZ) nanowire in PAN have a lot of positively charged oxygen vacancies with Lewis acid character, which may attract the anion of lithium salt and thus promote the dissociation of salts. The addition of YSZ nanoparticles or YSZ nanowires has a different degree of improvement in ionic conductivity compared to the absence of YSZ. The improvement effect of YSZ nanowires is better, and 7YSZ (7 mol% of Y_2_O_3_-doped ZrO_2_ nanowires) had a high room-temperature ionic conductivity of 1.07 × 10^−5^ S/cm at 30°C with an enhancement of two orders of magnitude compared with pristine PAN electrolyte (Figure 4B). Recently, Tao et al. (Sheng et al., 2018) incorporated Mg_2_B_2_O_5_ nanowires into PEO-LiTFSI-based solid electrolyte. The composite electrolytes exhibit good mechanical properties, outstanding electrochemical stability, and ionic conductivity, because of the fast ion motion on the surfaces of Mg_2_B_2_O_5_ and interactions between the Mg_2_B_2_O_5_ and TFSI^−^ (Figure 4C). In addition, other inert oxide ceramics have also been reported to improve the SPE performance, such as LiAlO_2_ (Gang et al., 1992; Hu et al., 2007), ZnO (Xiong et al., 2006), Fe_3_O_4_ (Reddy et al., 2006), and BaTiO_3_ (Itoh et al., 2003a,b).

## Solid Polymer Electrolytes With Fast-ion Conductive Ceramics

**Figure 5:**
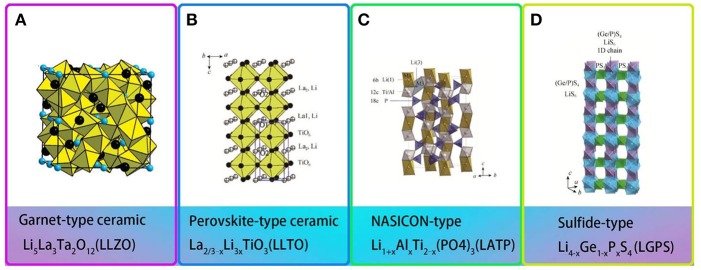
Structures of different types of fast ion conductors **(A)** Framework of garnet-type ceramic [Reproduction with permission from O'Callaghan et al. (2008) Copyright© 2008, American Chemical Society] **(B)** Crystal structure of perovskite-type ceramic. [Reproduction with permission from Stramare et al. (2003) Copyright© 2003, American Chemical Society] **(C)** Crystal structure of NASICON-type ceramic. [Reproduction with permission from Perez-Estebanez et al. (2014) Copyright© 2015, The Royal Society of Chemistry] **(D)** Crystal structure of Sulfide-type ceramic. [Reproduction with permission from Kamaya et al. (2011) Copyright© 2011, nature].

Fast ion conductor ceramics, also known as active inorganic electrolytes, exhibit a high ionic conductivity of up to 10^−2^ S/cm at 25°C. Four structures of fast ion conductors are displayed in Figure 5.

However, the poor interfacial contact restricts their direct use as solid electrolytes. Thus, composite of fast ion conductor ceramics with polymer can take full advantages of both parts. Fast ionic conductors commonly have garnet-type, NASICON-type and LISICON-type ceramics etc. Table 3 gives a summary of fast-ion conductive ceramics/polymer solid electrolytes.

**Table 3:** Summary of fast-ion conductive ceramics/polymer solid electrolytes.

**Year**	**Polymer electrolyte ingredients**	**Ionic conductivity (S/cm)**	**References**
2003	Li_5_La_3_M_2_O_12_ (M = Nb, Ta)-PEO-LiPF_6_	10^−6^ (25°C)	Ratner et al., 2000
2015	Li_7_La_3_Zr_2_O_12_(LLZO)-PEO-LiClO_4_	4.42 × 10^−4^ (55°C)	Shi et al., 2017
2015	Li_0.33_La_0.557_TiO_3_(LLTO) random nanowire-PAN-LiClO_4_	2.4 × 10^−4^ (25°C)	Subianto et al., 2009
2015	Li_2.5_Al_0.5_Ge_1.5_(PO_4_)_3_ (LAGP)-PEO-LiClO_4_	2.6 × 10^−4^ (55°C)	Tikekar et al., 2016
2015	Li_7_La_3_Zr_2_O_12_(LLZO)-PEO-LiClO_4_	4.42 × 10^−4^ (55°C)	Shi et al., 2017
2016	Li_6.4_La_3_Zr_2_Al_0.2_O_12_(LLZO)-PEO-LiTFSI	2.5 × 10^−4^ (25°C)	Stavila et al., 2014
2016	Li_1.5_Al_0.5_Ge_1.5_(PO_4_)_3_ (LAGP)-PEO-LiTFSI	6.76 × 10^−4^ (60°C)	Thokchom et al., 2008
2016	Li_10_GeP_2_S_12_-PEO-LiTFSI	10^−5^ (25°C)	Verma et al., 2010
2017	Li_6.20_Ga_0.30_La_2.95_Rb_0.05_Zr_2_O_12_-PVDF-LiTFSI	1.62 × 10^−3^ (25°C)	Scrosati and Garche, 2010
2017	Li_6.75_La_3_Zr_1.73_Ta_0.23_O_12_-PVDF-LiClO_4_	5 × 10^−4^ (25°C)	Siqueira and Ribeiro, 2006
2017	Li_0.33_La_0.557_TiO_3_(LLTO) aligned nanowire-PAN-LiClO_4_	6.05 × 10^−5^ (30°C)	Srivastava and Tiwari, 2009
2017	Li_1.3_Al_0.3_Ti_1.7_(PO_4_)_3_(LATP)-PEO-BPEG-LiTFSI	2.5 × 10^−4^ (60°C)	Thangadurai et al., 2003
2018	Li_7_La_3_Zr_2_O_12_ (LLZO)-PEO-LiTFSI	1.12 × 10^−4^ (25°C)	Quartarone and Mustarelli, 2011
2018	Li_6.4_La_3_Zr_1.4_Ta_0.6_O_12_(LLZTO)-PEO-LiTFSI	10^−4^ (55°C)	Sheng et al., 2017
2018	Li_0.33_La_0.557_TiO_3_-PEO-LiClO_4_	2.4 × 10^−4^ (25°C)	Stramare et al., 2003
2018	3D-Li_0.35_La_0.55_TiO_3_(LLTO)-PEO-LiTFSI	8.8 × 10^−5^ (25°C)	Sun et al., 2014

### Garnet-Type Composite Polymer Electrolytes

From the moment in 2007 when Li_7_La_3_Zr_2_O_12_ (LLZO) was first found, garnet-type Li solid-state electrolyte generates great interest in recent years. Li_7_La_3_Zr_2_O_12_ (LLZO), garnet-type Li solid-state electrolyte, has attracted much attention since it was first reported in 2007 (Xie H. et al., 2018). Li_5_La_3_M_2_O_12_ (M = Nb, Ta) is the first reported lithium ion conductor with a garnet structure (Figure 5A; Thangadurai et al., 2003; O'Callaghan et al., 2008). The traditional garnet chemical formula is A_3_B_2_(XO_4_)_3_ (A = Ca, Mg, Y, La or rare-earth elements; B = Al, Fe, Ga, Ge, Mn, Ni, or V). Garnet-type Li solid-state electrolyte has high ionic conductivity and wide electrochemical window (Wu et al., 2017). At room temperature, the ionic conductivity of Li_5_La_3_M_2_O_12_ (M = Nb, Ta) reached 10^−3^ S/cm and exhibits outstanding chemical stability over a wide temperature range. However, when the all-solid-state battery is assembled using garnet-type ceramics, the electrode/electrolyte interface always shows poor conductivity, resulting in deteriorated battery performance, as well as increased interface resistance and decreased ionic conductivity (Chen et al., 2018). Polymer/Garnet composite electrolytes offer an option of improving the overall electrochemical performances.

With a large specific surface area, nanoscale garnet ceramic fillers improve the transition rate of ions (Kumar and Scanlon, 2000). A composite electrolyte composed of PEO containing 52.5 wt.% Li_7_La_3_Zr_2_O_12_(LLZO) particles displays a conductivity which reaches 4.42 × 10^−4^ S/cm at 55°C (Thokchom et al., 2008). Li_6.75_La_3_Zr_1.75_Ta_0.25_O_12_ (LLZTO) is selected as an active filler and dispersed into PVDF matrix to fabricate PVDF/LLZTO hybrid electrolytes (Zhang X. et al., 2017). The hybrid electrolyte with 10 wt.% LLZTO loadings exhibited the highest ionic conductivity (5 × 10^−4^ S/cm), about seven times more than none LLZTO. It is attributed to that LLZTO particles react with Li^+^ via acid-base interaction. Dissociation of the lithium salt will raise the carrier density for conduction. Furthermore, the garnet ceramic filler contributes to reduce the crystallinity of polymer and so to increase the ionic conductivity. Instead of simply mixing active ceramic particles into polymers, Goodenough et al. (Chen et al., 2018) introduced a novel approach of composite polymer into ceramic.

As a consequence, high ionic conductivity (10^−4^ S/cm at 55°C) were gained and the electrochemical window of 0–5.0 V. As used in the all-solid-state Li/LiFePO_4_ cells, both “ceramic-in-polymer” and “polymer-in-ceramic” with a LiTFSI salt display remarkable cycling stability. The systems, “polymer-in-ceramic” provide higher mechanical strength and safety than “ceramic-in-polymer.”

**Figure 6:**
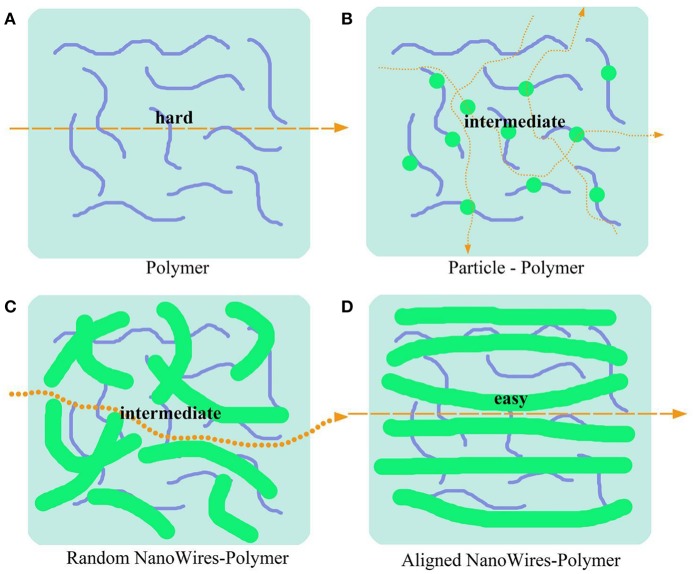
Schematic of conduction mechanism in three kinds of morphologies solid polymer electrolytes. **(A)** Ionic transport path in pure polymer electrolytes. **(B–D)** Ionic transport path in polymer-based composite electrolytes with nanoparticles **(B)**, random nanowires **(C)** and aligned nanowires **(D)**.

Morphologies of ceramics fillers such as particles, distribution of nanowire and 3D framework may affect the ionic conductivity of polymer composite electrolytes. Unlike particles and random nanowires, aligned nanowires combined with polymers can provide continuous transport pathways for Li^+^ (Figure 6). Cui et al. (Liu et al., 2017) compares the different morphologies of LLZO to evaluate their benefits for ionic transport. They found that a composite polymer electrolyte with well-aligned inorganic nanowires (LLZO) shows an ionic conductivity of 6.05 × 10^−5^ S/cm at 30°C, which was increased by almost one order of magnitude than the composite with randomly aligned nanowires or nanoparticles. The appreciable conductivity improvement is due to Li^+^ migration without crossing junctions on the nanowire surfaces.

In addition to 1D nanowires (Bae et al., 2018), prepared 3D ceramic Li_6.28_La_3_Zr_2_Al_0.24_O_12_ networks by using hydrogel and mixed it into polymer solution to attain solid electrolyte. The designed structure is believed to have high conductivity (8.5 × 10^−5^ S/cm at 25°C) and good interfacial compatibility with electrodes. The integrated structure of 3D LLZO structure provides continuous 3D network of conduction pathways leading to highly improved ionic conductivity and mechanical properties. Similarly, 3D garnet nanofiber networks-polymer composite was also prepared (Fu et al., 2016). In this approach, the LLZO porous structure, composed of casually distributed and interconnected nanofibers, forms a continuous transport network for Li^+^. The LiTFSI-PEO polymer is then filled into the porous 3D LLZO ceramic networks, forming the 3D garnet-polymer composite films. Then LiTFSI-PEO polymer and porous 3D Inorganic structure are combined to synthesize a 3D LLZO-polymer composite membrane which exhibited a high ionic conductivity of 2.5 × 10^−4^ S/cm at 25°C. The three-dimensional ion transport network offers a new option of designing composite electrolytes.

### Perovskite-Type Composite Polymer Electrolytes

**Figure 7:**
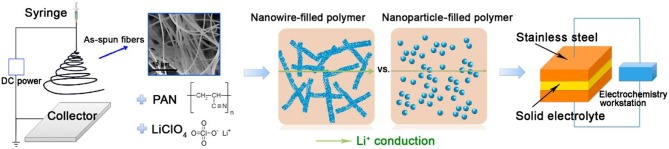
Schematic illustration for the synthesis of ceramic nanowire-filled polymer-based composite electrolytes. [Reproduction with permission from Liu et al. (2015) Copyright© 2009, American Chemical Society].

Perovskite-type solid electrolytes Li_3x_La_2/3−x_TiO_3_ (LLTO) has a cubic structure with space group of P4/mmm and C-mmm (Figure 5B; Stramare et al., 2003). LLTO is well-known for its stable at high voltages. However, its preparation conditions are very strict and the ionic conductivity is also low. Recently, a polymer–ceramic composite electrolyte PEO/LiClO_4_ has been studied by composite PEO with Li_0.33_La_0.557_TiO_3_ nanowires. It exhibited extreme lithium-ion conductivity of 2.4 × 10^−4^ S/cm at 25°C (Zhu et al., 2018). Cui et al. (Liu et al., 2015) studied the effect of two different morphological LLTO materials on the ionic conductivity of polymer electrolytes, which are nanoscale particles and nanowire LLTO, respectively (Figure 7). The introduction of LLTO nanowire into PAN achieved higher ionic conductivity 2.4 × 10^−4^ S/cm at room temperature as compared to pristine PAN film. The composite electrolyte offers a 3D long distance Lithium-ion transmission network, which reduce the negative effect of agglomeration of inorganic ceramics in polymers relative to nanoparticles. This work opened a new way to develop one-dimensional fast ion conductive ceramic materials in solid electrolytes for lithium batteries.

The ionic conductivity has a strong relationship with the ceramic component loadings in the composite electrolyte. Generally, the higher the content, the lower the ionic conductivity will be, because nano-sized ceramic fillers are agglomerated and may block the percolation network around the phase interface. Meanwhile, in order to achieve high security of the composite electrolyte, it is necessary to reduce the proportion of combustible organic polymer content and increase the flame-retardant inorganic ceramic portion. Goodenough et al. (Bae et al., 2018) constructed a 3D-LLTO/PEO composite electrolyte using a hydrogel-derived method. The LLTO was incorporated into the hydrogel template, then it was cast with PEO after removing the template. This artificial 3D infiltration network naturally avoids the agglomeration of nanofillers compared to the traditional simple dispersion process, and its ultra-high specific surface area provides a continuous phase interface network as lithium ion transport channel. Therefore, this composite electrolyte displayed a high ionic conductivity of 8.8 × 10^−5^ S cm^−1^ at room temperature.

### NASICON-Type Composite Polymer Electrolytes

NASICON-type ceramics (aka “sodium super ion conductor”) were firstly discovered in 1968 with composition of NaM_2_(PO_4_)_3_ (M = Ge, Ti, Zr) (Epp et al., 2015). Surdrean et al. firstly reported NASICON-type solid electrolyte LiZr_2_(PO_4_)_3_ at 1989. For formula LiM_2_(XO_4_)_3_, [M_2_(XO_4_)_3_] constitutes the basic structure of NASICON. The MO_6_ octahedron and the XO_4_ tetrahedron are connected in a common angle to form Li-ion transmission channel. Aono et al. (1990) first reported doped trivalent ions into LiTi_2_(PO_4_)_3_ and found that the ionic conductivity was improved. In 2014, Perez-Estebanez et al. (2014) achieved high conductivity in the Li_1+x_Al_*x*_Ti_2−x_(PO_4_)_3_ (LATP) of 6.76 × 10^−4^ S/cm at 60°C (Figure 5C). After that, research on NASICON-type electrolyte experienced fast growth because of its high ionic conductivity (over 10^−3^ S/cm) at ambient temperature and stable in the ambient atmosphere.

**Figure 8:**
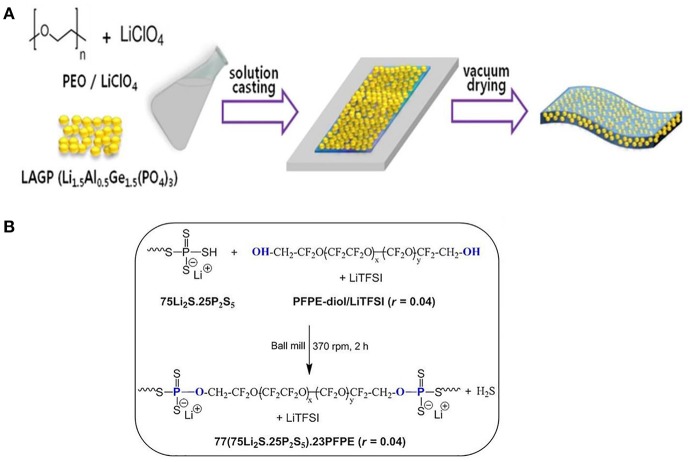
**(A)** Schematic presentation of preparation for PEO-LiClO4-LAGP hybrid solid electrolyte. [Reproduction with permission from Jung et al. (2015) Copyright 2017, The Electrochemical Society.] **(B)** Synthesis of the hybrid electrolyte 77(75Li_2_S.25P_2_S_5_).23PFPE (*r* = 0.04) by mechanochemical reaction. [Reproduction with permission from Villaluenga et al. (2016). Copyright 2016, National Academy of Sciences].

Pan group (Yang et al., 2017) fabricated Li_1.3_Al_0.3_Ti_1.7_(PO_4_)_3_- PEO polymer electrolyte. The discharge specific capacity of LiFePO_4_/Li using this polymer electrolyte was 158.2 and 94.2 mAh/g at 0.1 and 2 C, respectively. LATP can not only form pathways for lithium transportation in the interphase, leading to improved ionic conductivity, but also physically resist lithium dendrite growth. Lithium aluminum germanium phosphate (LAGP) is also a kind of NASICON-type fast ion conductor ceramic with relative high ionic conductivity (>10^−4^ S/cm). Zhao et al. (2016a) similarly incorporated NASICON-type Li_1.5_Al_0.5_Ge_1.5_(PO_4_)_3_ (LAGP) as Li^+^ conductors into PEO matrix. The resultant polymer electrolyte displayed a wide electrochemical window of 0–5.3 V and an ion-conductivity of 6.76 × 10^−4^ S/cm at 60°C. More intriguingly, such polymer electrolyte based LiFePO_4_/Li battery showed prominent cycling stability (90% after 50 cycles). Jung et al. (2015) designed a stretchable ceramic-polymer composite electrolyte membrane where NASICON-type LAGP were incorporated into a polymer-Li salt LiCLO_4_ matrix, to synthesize a polyethylene oxide solid electrolyte membrane (Figure 8A). The PEO-LiCLO_4_-LAGP composite electrolyte with 60–80 wt.% LAGP is still capable of providing enough mechanical modulus and good electrochemical performance. Li/LiFePO_4_ cells initial discharge capacities reach 138.5 mAh/g and deliver good capacity retention.

### Sulfide-Type Polymer Electrolytes

Sulfide-type electrolytes show supreme ion-conductivities in the magnitude of 10^−2^ S/cm at room temperature (Kamaya et al., 2011). However, they demonstrate instability due to reaction with water vapor in air. Sulfide-type ceramics can be divided into three categories: glasses, glass-ceramic, and ceramic. The entire types ion-conductivity can near or exceed liquid electrolyte. Glass/glass-ceramic Li_2_S-P_2_S_5_ and ceramic thio-LISICON Li_4−x_Ge_1−x_P_x_S_4_ (0 < *x* < 1) are the most promising ones. Li_10_GeP_2_S_12_ and PEO has been composited to prepare solid electrolyte membrane (Zhao et al., 2016b). The conductivity at room temperature reaches 10^−5^ S/cm, which is higher than other conventional PEO electrolyte at least one order of magnitude, and the electrochemical window spans between 0 and 5.7 V. It greatly expands the selection range of positive electrode materials and presents improved stability to lithium metal. The solid polymer batteries show capacity retentions approaching 92.5% after 50 cycles. Villaluenga et al. (2016) prepared a non-flammable composite electrolytes by fully mechanochemical reaction between hydroxy-terminated perfluoropolyether (PFPE-diol), LiTFSI and 75Li_2_S·25P_2_S_5_ by ball milling for 2 h. The electrolyte containing 77 wt.% (75Li_2_S·25P_2_S_5_) and 23 wt.% PFPE-diol/LiTFSI displays a conductivity of 10^−4^ S/cm at room temperature (Figure 8B).

### Solid Polymer Electrolytes With Ionic Liquid

An ionic liquid (IL) is a molten salt at low temperatures and generally consist of organic cations and inorganic anions (Zhao et al., 2016b). Due to the special state, ionic liquids have the characteristics of vapor pressure free, high electrochemical stability, and good thermal stability (Armand et al., 2009). Although ionic liquids have high ionic conductivity, they are not suitable for direct use as electrolytes because of low viscosity. The combination of ionic liquid and polymer offers an option as solid electrolyte for lithium ion batteries.

The introduction of IL into the polymer results in higher ionic conductivity, but it is usually accompanied by a decrease in mechanical strength, especially at high temperature. Lower IL concentration leads to higher mechanical strength, and a smoother continuous electrolyte surface, which is more favorable for ion transport. Therefore, the amount of IL plays an important influence on the ionic conductivity and mechanical properties. Moreover, battery cycling at high temperatures usually causes decomposition of IL components, resulting in degraded performances. It added one more requirement of the polymer components to retain high IL contents.

**Figure 9:**
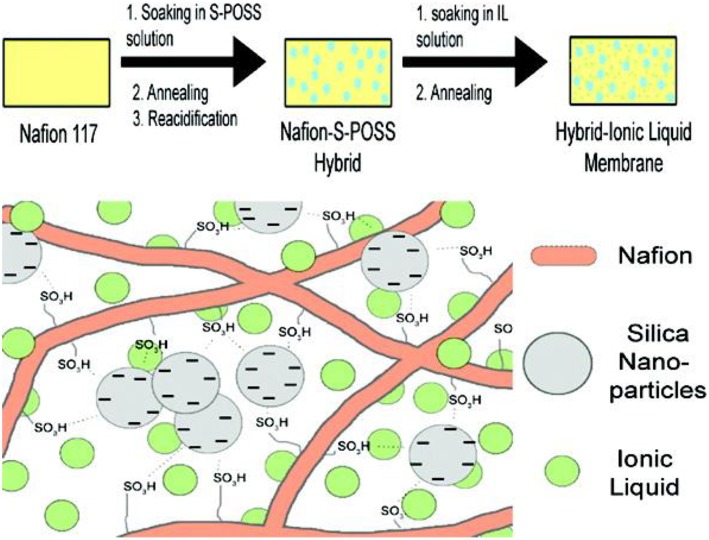
Schematic illustration of the overall procedure for the preparation of Hybrid-Ionic liquid electrolyte. [Reproduction with permission from Subianto et al. (2009). Copyright© 2009, American Chemical Society].

IL-based polymer electrolytes are mainly classified into three categories. (1) polymer doped IL; (2) ILs/polymerizable monomers crosslinks; (3) polymeric ionic liquids (PILs). The first one is just IL added to the polymer solution or infused in the polymer film directly. For example, Subianto et al. (2009) prepared an electrolyte consisting of IL, silica nano-particles, and Nafion by using sulfonated polyhedral oligomeric silsesquioxane (S-POSS) modified Nafion membranes soaking with 1-butyl-3-methylimidazolium bis- (trifluoromethylsulfonyl)imide (BMI-BTSI) (Figure 9). The thermal stability of Nafion films was improved after ionic liquid infiltration. More importantly, the conductivity of the infiltrated films is increased by one to two orders of magnitude than that of the unmodified one. ILs/polymer monomer cross-linking is the mixing of ILs and polymerizable monomers to obtain electrolytes by means of thermal or photo polymerization. Polymeric ionic liquids (PILs) can be designed by the direct polymerization of polymerizable IL-based monomer or polymerizing a modified polymer and an IL monomer. By taking full advantages of the specific properties of ionic liquids and polymers, PIL membrane has generated great interest in recent years. By adopting solution cast technique, Karuppasamy et al. (2016) designed PIL synthesized solid electrolytes by preparing ionic liquids of lithium N, N-bis(trifluoromethanesulfonyl)imide (LiTFSI) in N-ethyl-N-methylimidazolium–bis(trifluoromethanesulfonyl) imide (EMImTFSI) IL with incorporated organic solvent and nanoparticle into PEO. The prepared PIL electrolyte exhibits high ionic conductivity of 10^−2^ S/cm and high electrochemical stability. Yang et al. (Li et al., 2011) designed a solid electrolyte by combining PIL with different anions such as ${\text{BF}}_{4}^{-}$, ${\text{PF}}_{6}^{-}$, ${\text{ClO}}_{4}^{-}$, and $\text{N}{\left({\text{CF}}_{3\;}\;{\text{SO}}_{2}\right)}_{2}^{-}$. PILs electrolyte with 1g2-MA-BF_4_/LiBF_4_ exhibited ionic conductivity as high as 1.35 × 10^−4^ S/cm at 30°C. Starting from PEO, modified sepiolite (TPGS-S), LiTFSI, and 1-Butyl-1-methylpyrrolidinium bis(trifluoromethanesulfonyl)imide (PYR14TFSI) ionic liquid, electrolytes was synthesized via solvent free extrusion method (Gonzalez et al., 2018). The resultant polymer electrolyte displayed wide electrochemical window of 4.2 V and ion-conductivity of 5× 10^−4^ S/cm at 60°C.

## Solid Polymer Electrolytes With MOFs

**Figure 10:**
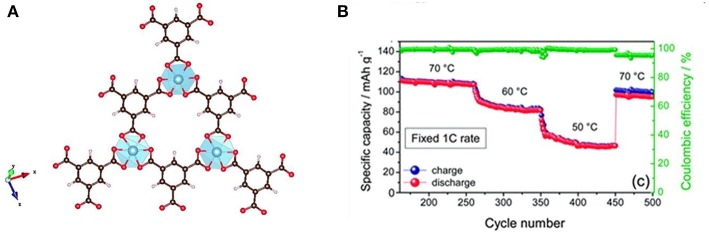
**(A)** Schematic diagram of the ideal network structure of an aluminum(III)-1,3,5-benzenetricarboxylate (Al-BTC) metal organic framework. **(B)** Electrochemical characteristics of the LiFePO_4_/S4-NCPE/Li cell at different temperatures and current regimes. [Reproduction with permission from Gerbaldi et al. (2014). Copyright© 2014, The Royal Society of Chemistry].

**Figure 11:**
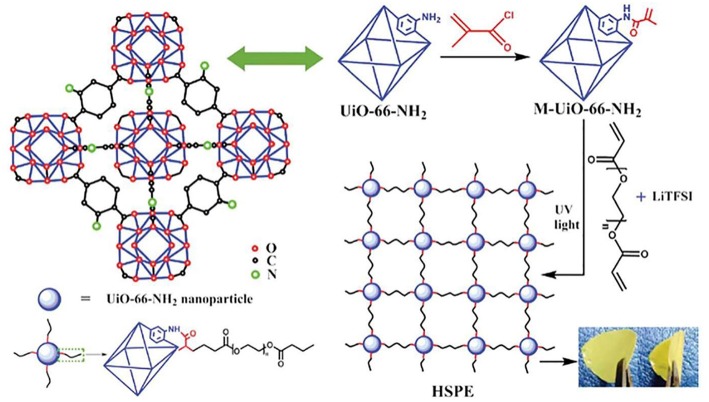
Synthetic route of the hybrid covalently linked MOF-PEGDA-based all-solid-state electrolyte. [Reproduction with permission from Wang Z. et al. (2018). Copyright© 2018, The Royal Society of Chemistry].

Metal–organic frameworks (MOFs) are a new kind of porous material, which are composed of metal ions and bridging organic ligands (Stavila et al., 2014; Indra et al., 2018; Xie X. C. et al., 2018). MOFs have many properties such as porosity, large specific surface area, and polymetallic sites (Yuan et al., 2013), so they are widely used in many fields including gas adsorption, molecular separation, drug delivery (Mueller et al., 2006; Kuppler et al., 2009; Li et al., 2009). Many investigations have indicated that MOFs also has positive effect on increased ionic conductivity due to high specific surface and the good adsorption property. Yuan et al. (2013) prepared a new SPE by the addition of Zn-based MOF-5 into PEO polymer electrolyte. The combination of MOFs and polymer showed positive effect on the mechanical and electrochemical properties as solid electrolyte. The ionic conductivity of these membrane can reach 3.16 × 10^−5^ S/cm at ambient temperature, which is attributed to two parts. Firstly, the interaction of the Lewis acidic sites on the MOF-5 with the PEO chain and the lithium salt hinder the crystallization of the PEO, and facilitate the formation of Li ^+^ conductive channels. Secondly, the isotropic open MOF-5 can adsorb solvent to accelerate the transport of ions. Gerbaldi et al. (2014) proposed a new filler material (aluminum-based MOF) (Figure 10A), which was successfully prepared and incorporated in a PEO-based polymer matrix. Ionic conductivity of the composite membrane is two orders of magnitude greater than that without mixed MOFs. lithium batteries (Li/LiFePO_4_) with the electrolyte showed distinguished charge-discharge performance and high specific capacity. At 1 C rate, the battery can still cycle stably at 50°C, and the decay of specific capacity is not obvious when restored to 70°C. After 500 cycles, the capacity is almost maintained as the initial, and the Coulombic efficiency is only slightly decreased. This shows an excellent capacity retention capability and good cycle stability (Figure 10B). Recently, Wang Z. et al. (2018) synthesized a new chemically linked composite MOF-polymer electrolyte. The film was prepared by photopolymerization with post-synthetic modification of the MOF (M-UiO-66-NH_2_), poly(ethylene glycol) diacrylate and LITFSI (Figure 11). The interface between MOF and polymer provides a fast channel for lithium-ion transport, accordingly the conductivity of the composite electrolyte (HSPE-1-8) is 4.31 × 10^−5^ S/cm at 30°C that is up to five times more than that of no composite MOF. The solid Li/LiFePO_4_ cells assembled with these SPEs cycled at 60°C demonstrated excellent coulombic efficiencies.

## Solid Polymer Electrolytes With Cellulose

Cellulose is a non-toxic harmless, inexpensive, and natural eco-friendly materials with high mechanical strength and a large specific surface area (Baxter et al., 2009; Sheng et al., 2017) Due to the unique properties, cellulose can not only enhance the mechanical properties of polymers in electrolytes, but hinder the growth of lithium dendrites effectively acting as a physical barrier. The interface between cellulose and polymer behaves as a channel for ion transport, facilitating ion transport. In addition, polar groups in cellulose can improve salt dissociation (Shi et al., 2017). Nair et al. (2009) reported a polymer composite electrolyte with cellulose reinforcement. The reinforced electrolyte exhibited a high ionic conductivity (2.0 × 10^−4^ S/cm at 25°C) and an exceptional mechanical property, which is expected for flexible electronic devices applications. Furthermore, ionic liquid compounded with cellulose can solve the issues of IL leakage in the composite electrolyte. Shi et al. (2017) designed a new type of 3D self-assembled polymeric ionic liquid (PIL)-nano-cellulose to form polymer electrolyte. The structure not only enhances the mechanical properties of the SPE, but also forms strong lithium coordination to promote lithium salt dissolution. The dissolved lithium salt can combine with IL to form an ion-conducting domain, thereby promoting ion transport. Asghar et al. (2012) adequately utilizes the characteristics of networked cellulose (NC), with mechanical strength and adopted it to design quasi-solid PEG-LiClO4-NC polymer electrolyte. The resultant composite electrolyte with a 12.8 wt.% NC resulted in the highest ion conductivity (10^−4^ S/cm at 25°C) and is electrochemically stable up to 4.7 V. Similarly, Zhang et al. (2014) combined cellulose non-woven with PCA-PEO to fabricate a rigid-flexible coupling SPE, upgrading their comprehensive properties of the composite electrolyte significantly.

## Summary and Outlook

Although lithium-ion batteries have long been commercialized, the use of liquid electrolytes has some disadvantages such as poor safety and unstable electrochemical performance, which greatly limits its further development and wider applications. Solid composite polymer electrolyte in lithium-ion batteries has received a lot of attention lately because of its low flammability, good flexibility, excellent thermal stability, and high safety. In this review, we have provided fundamental understandings of the ionic conductivity mechanisms and interfaces for solid composite electrolytes, in the meantime, recent progresses on polymer-based composite electrolytes were summarized, including polymer/inert ceramics, polymer/fast-ion conductive, polymer/ionic liquid, polymer/MOFs, and polymer/cellulose composite electrolytes.

Although substantial researches have been dedicated to the polymer-based composite electrolytes, some fundamental issues still need to be solved urgently before commercialization. For example, ionic conductivity of the composite solid electrolyte still differs by several orders of magnitude from the liquid counterpart; many polymer-based solid electrolytes exhibit high ionic conductivity at high temperatures, while it drops dramatically at lower temperatures; the conductivity mechanism and interfacial interaction need to be further clarified not accelerate further studies.

At present, the polymer/ionic liquid solid electrolyte inevitably causes a decrease in mechanical properties when obtaining high ionic conductivity, which has great safety hazards. The difficulty in polymer/inert ceramic solid electrolytes is how to construct a good dispersion and strengthen the interaction between the filler and the polymer, which restricts the further improvement of ionic conductivity. In comparison, polymer/fast ion conductors composite electrolytes have both high ionic conductivity at room temperature and good mechanical properties. The future development direction of polymer-based solid electrolytes is likely to be the combination of fast ion conductors and polymers, which can combine the advantages of high ion conductivity of fast ion conductors and solve the problem of poor interface contact. Of all the types of polymer-based composite solid electrolytes, SPEs with fast ion conductors have gained all the advantages and are the direction of development of commercial solid electrolytes.

The following aspects were recommended of focusing on solid electrolyte in future developments. Firstly, using materials genome database to analyze, guide, and design composite material can promote efficiency and cost savings. Material calculations facilitate an in-depth understanding of the material. The corresponding ionic mechanism can be simulated and interpreted by material calculations. Secondly, most of battery materials, such as electrodes, electrolytes, and SEI films, are sensitive to electron beams and difficult to observe under conventional transmission electron microscopy (TEM). Advanced characterization techniques can facilitate analysis of material mechanisms. For example, using cryo-electron microscopy, Zachman et al. (2018) reveals atomic structure of sensitive battery materials and interfaces. Similarly, Meng et al. (Wang et al., 2017) studied the interface of solid electrolytes via Cryogenic TEM, which has greatly promoted further research on the interface. Finally, struggling to find suitable composite electrolytes with high conductivities at low temperature always deserves more study. Commercial solid electrolytes require high ionic conductivity at room temperature, safety and easy processing to compete with the liquid counterpart.

## Author Contributions

PY, JW, and HY collected the data, performed the statistical analysis, interpretation of the results, and wrote the manuscript. ZD, YL, and JL carried out the bibliographical research and performed the inferential analyses together with other authors. ML and XL offered Suggestions and revisions in English for the article.

### Conflict of Interest Statement

The authors declare that the research was conducted in the absence of any commercial or financial relationships that could be construed as a potential conflict of interest.

## References

[B1] Agrawal R. C. Pandey G. P. (2008). Solid polymer electrolytes: materials designing and all-solid-state battery applications: an overview. J Phys D Appl Phys. 41:223001 10.1088/0022-3727/41/22/223001

[B2] Aliahmad N. Shrestha S. Varahramyan K. Agarwal M. (2016). Poly(vinylidene fluoride-hexafluoropropylene) polymer electrolyte for paper-based and flexible battery applications. AIP Adv. 6:065206 10.1063/1.4953811

[B3] Aono H. Sugimoto E. Sadaoka Y. Imanaka N. Adachi G. (1990). Ionic-conductivity of solid electrolytes based on lithium titanium phosphate. J. Electrochem. Soc. 137, 1023–1027. 10.1149/1.2086597

[B4] Aravindan V. Vickraman P. (2008). Characterization of SiO_2_ and Al_2_O_3_ incorporated PVdF-HFP based composite polymer electrolytes with LiPF3(CF3CF2)(3). J. Appl. Polym. Sci. 108, 1314–1322. 10.1002/app.27824

[B5] Armand M. Endres F. MacFarlane D. R. Ohno H. Scrosati B. (2009). Ionic-liquid materials for the electrochemical challenges of the future. Nat. Mater. 8, 621–629. 10.1038/Nmat2448 19629083

[B6] Asghar A. Samad Y. A. Lalia B. S. Hashaikeh R. (2012). PEG based quasi-solid polymer electrolyte: mechanically supported by networked cellulose. J. Memb. Sci. 421, 85–90. 10.1016/j.memsci.2012.06.037

[B7] Bae J. Li Y. Zhang J. Zhou X. Zhao F. Shi Y.. (2018). A 3D nanostructured hydrogel-framework-derived high-performance composite polymer Lithium-ion electrolyte. Ange. Chem. Int. Ed. 57, 2096–2100. 10.1002/anie.201710841 29314472

[B8] Baxter J. Bian Z. Chen G. Danielson D. Dresselhaus M. S. Fedorov A. G. (2009). Nanoscale design to enable the revolution in renewable energy. Energy Environ. Sci. 2, 559–588. 10.1039/b821698c

[B9] Camacho-Forero L. E. Balbuena P. B. (2018). Exploring interfacial stability of solid-state electrolytes at the lithium-metal anode surface. J. Power Sources 396, 782–790. 10.1016/j.jpowsour.2018.06.092

[B10] Capuano F. Croce F. Scrosati B. (1991). Composite polymer electrolytes. J. Electrochem. Soc. 138, 1918–1922. 10.1149/1.2085900

[B11] Chen L. Li Y. Li S.-P. Fan L.-Z. Nan C.-W. Goodenough J. B. (2018). PEO/garnet composite electrolytes for solid-state lithium batteries: from ceramic-in-polymer to polymer-in-ceramic. Nano Energy 46, 176–184. 10.1016/j.nanoen.2017.12.037

[B12] Chen R. J. Qu W. J. Guo X. Li L. Wu F. (2016). The pursuit of solid-state electrolytes for lithium batteries: from comprehensive insight to emerging horizons. Mater. Horizon. 3, 487–516. 10.1039/c6mh00218h

[B13] Croce F. Appetecchi G. B. Persi L. Scrosati B. (1998). Nanocomposite polymer electrolytes for lithium batteries. Nature 394, 456–458.

[B14] Cui Y. Baker A. P. Xu X. Xiang Y. Wang L. Lavorgna M. (2015). Enhancement of Nafion based membranes for direct methanol fuel cell applications through the inclusion of ammonium-X zeolite fillers. J. Power Sources 294, 369–376. 10.1016/j.jpowsour.2015.06.078

[B15] Cui Y. Liu Y. Wu J. Zhang F. Baker A. P. Lavorgna M. (2018). Porous silicon-aluminium oxide particles functionalized with acid moieties: an innovative filler for enhanced Nafion-based membranes of direct methanol fuel cell. J. Power Sources 403, 118–126. 10.1016/j.jpowsour.2018.09.090

[B16] Dias F. B. Plomp L. Veldhuis J. B. J. (2000). Trends in polymer electrolytes for secondary lithium batteries. J. Power Sources 88, 169–191. 10.1016/s0378-7753(99)00529-7

[B17] Do J. S. Chang C. P. Lee T. J. (1996). Electrochemical properties of lithium salt-poly(ethylene oxide)ethylene carbonate polymer electrolyte and discharge characteristics of Li/MnO_2_. Solid State Ionics 89, 291–298. 10.1016/0167-2738(96)00343-8

[B18] Dong X. C. Wang L. (2005). Compositions, structures and properties of polymer electrolytes for lithium ion battery. Progr. Chem. 17, 248–253.

[B19] Epp V. Ma Q. Hammer E.-M. Tietz F. Wilkening M. (2015). Very fast bulk Li ion diffusivity in crystalline Li_1.5_Al_0.5_Ti_1.5_(PO_4_)(3) as seen using NMR relaxometry. Phys. Chem. Chem. Phys. 17, 32115–32121. 10.1039/c5cp05337d 26580669

[B20] Farrington G. C. Briant J. L. (1979). Fast ionic transport in solids. Science 204, 1371–1379. 10.1126/science.204.4400.1371 17814181

[B21] Fergus J. W. (2010). Ceramic and polymeric solid electrolytes for lithium-ion batteries. J. Power Sources 195, 4554–4569. 10.1016/j.jpowsour.2010.01.076

[B22] Fonseca C. P. Neves S. (2002). Characterization of polymer electrolytes based on poly(dimethyl siloxane-co-ethylene oxide). J. Power Sources 104, 85–89. 10.1016/s0378-7753(01)00902-8

[B23] Forsyth M. Tipton A. L. Shriver D. F. Ratner M. A. MacFarlane D. R. (1997). Ionic conductivity in poly(diethylene glycol-carbonate)/sodium triflate complexes. Solid State Ionics 99, 257–261. 10.1016/s0167-2738(97)00115-x

[B24] Fu K. K. Gong Y. Dai J. Gong A. Han X. Yao Y.. (2016). Flexible, solid-state, ion-conducting membrane with 3D garnet nanofiber networks for lithium batteries. Proc. Natl. Acad. Sci. USA. 113, 7094–7099. 10.1073/pnas.1600422113 27307440 PMC4932948

[B25] Gang W. Roos J. Brinkmann D. Capuano F. Croce F. Scrosati B. (1992). Comparison of NMR and conductivity in (PEO)(8)LiCLO_4_+GAMMA-LiALO_2_. Solid State Ionics 53, 1102–1105. 10.1016/0167-2738(92)90297-3

[B26] Gerbaldi C. Nair J. R. Kulandainathan M. A. Kumar R. S. Ferrara C. Mustarelli P. (2014). Innovative high performing metal organic framework (MOF)-laden nanocomposite polymer electrolytes for all-solid-state lithium batteries. J. Mater. Chem. A 2, 9948–9954. 10.1039/c4ta01856g

[B27] Gonzalez F. Tiemblo P. Garcia N. Garcia-Calvo O. Fedeli E. Kvasha A.. (2018). High performance polymer/ionic liquid thermoplastic solid electrolyte prepared by solvent free processing for solid state lithium metal batteries. Membranes 8:55. 10.3390/membranes8030055 30072669 PMC6160972

[B28] Hu L. Tang Z. Zhang Z. (2007). New composite polymer electrolyte comprising mesoporous lithium aluminate nanosheets and PEO/LiClO4. J. Power Sources 166, 226–232. 10.1016/j.jpowsour.2007.01.028

[B29] Indra A. Song T. Paik U. (2018). Metal organic framework derived materials: progress and prospects for the energy conversion and storage. Adv. Mater. 30:1705146. 10.1002/adma.201705146 29984451

[B30] Itoh T. Ichikawa Y. Uno T. Kubo M. Yamamoto O. (2003a). Composite polymer electrolytes based on poly(ethylene oxide), hyperbranched polymer, BaTiO_3_ and LiN(CF_3_SO_2_)(2). Solid State Ionics 156, 393–399. 10.1016/s0167-2738(02)00682-3

[B31] Itoh T. Miyamura Y. Ichikawa Y. Uno T. Kubo M. Yamamoto O. (2003b). Composite polymer electrolytes of poly(ethylene oxide)/BaTiO_3_/Li salt with hyperbranched polymer. J. Power Sources 119, 403–408. 10.1016/s0378-7753(03)00261-1

[B32] Jung Y.-C. Lee S.-M. Choi J.-H. Jang S. S. Kim D.-W. (2015). All solid-state lithium batteries assembled with hybrid solid electrolytes. J. Electrochem. Soc. 162, A704–A710. 10.1149/2.0731504jes

[B33] Kamaya N. Homma K. Yamakawa Y. Hirayama M. Kanno R. Yonemura M.. (2011). A lithium superionic conductor. Nat. Mater. 10, 682–686. 10.1038/nmat3066 21804556

[B34] Karuppasamy K. Rhee H. W. Reddy P. A. Gupta D. Mitu L. Polu A. R. (2016). Ionic liquid incorporated nanocomposite polymer electrolytes for rechargeable lithium ion battery: a way to achieve improved electrochemical and interfacial properties. J. Ind. Eng. Chem. 40, 168–176. 10.1016/j.jiec.2016.06.020

[B35] Keller M. Appetecchi G. B. Kim G.-T. Sharova V. Schneider M. Schuhmacher J. (2017). Electrochemical performance of a solvent-free hybrid ceramic-polymer electrolyte based on Li_7_La_3_Zr_2_O_12_ in P(EO)(15)LiTFSI. J. Power Sources 353, 287–297. 10.1016/j.jpowsour.2017.04.014

[B36] Ketabi S. Lian K. (2013). Effect of SiO2 on conductivity and structural properties of PEO-EMIHSO4 polymer electrolyte and enabled solid electrochemical capacitors. Electrochim. Acta 103, 174–178. 10.1016/j.electacta.2013.04.053

[B37] Knauth P. (2009). Inorganic solid Li ion conductors: An overview. Solid State Ionics 180, 911–916. 10.1016/j.ssi.2009.03.022

[B38] Kumar B. Scanlon L. G. (2000). Composite electrolytes for lithium rechargeable batteries. J. Electroceramics 5, 127–139. 10.1023/a:1009958118260

[B39] Kuppler R. J. Timmons D. J. Fang Q.-R. Li J.-R. Makal T. A. Young M. D. (2009). Potential applications of metal-organic frameworks. Coord. Chem. Rev. 253, 3042–3066. 10.1016/j.ccr.2009.05.019

[B40] Li J.-R. Kuppler R. J. Zhou H.-C. (2009). Selective gas adsorption and separation in metal-organic frameworks. Chem. Soc. Rev. 38, 1477–1504. 10.1039/b802426j 19384449

[B41] Li M. Yang L. Fang S. Dong S. (2011). Novel polymeric ionic liquid membranes as solid polymer electrolytes with high ionic conductivity at moderate temperature. J. Memb. Sci. 366, 245–250. 10.1016/j.memsci.2010.10.004

[B42] Liang B. Tang S. Jiang Q. Chen C. Chen X. Li S. (2015). Preparation and characterization of PEO-PMMA polymer composite electrolytes doped with nano-Al2O3. Electrochim. Acta 169, 334–341. 10.1016/j.electacta.2015.04.039

[B43] Lin D. Liu W. Liu Y. Lee H. R. Hsu P.-C. Liu K.. (2016). High ionic conductivity of composite solid polymer electrolyte via *in situ* synthesis of monodispersed SiO_2_ nanospheres in poly(ethylene oxide). Nano Lett. 16, 459–465. 10.1021/acs.nanolett.5b04117 26595277

[B44] Lin D. Yuen P. Y. Liu Y. Liu W. Liu N. Dauskardt R. H.. (2018). A silica-aerogel-reinforced composite polymer electrolyte with high ionic conductivity and high modulus. Adv. Mater. 30:e1802661. 10.1002/adma.201802661 29939433

[B45] Ling S.-G. Peng J.-Y. Yang Q. Qiu J.-L. Lu J.-Z. Li H. (2018). Enhanced ionic conductivity in LAGP/LATP composite electrolyte. Chin. Phy. B, 27:038201 10.1088/1674-1056/27/3/038201

[B46] Liu J. Xu J. Y. Lin Y. Li J. Lai Y. Q. Yuan C. F. (2013). All-solid-state lithium ion battery: research and industrial prospects. Acta Chim. Sin. 71, 869–878. 10.6023/a13020170

[B47] Liu W. Lee S. W. Lin D. Shi F. Wang S. Sendek A. D. (2017). Enhancing ionic conductivity in composite polymer electrolytes with well-aligned ceramic nanowires. Nat. Energy 2:17035 10.1038/nenergy.2017.35

[B48] Liu W. Lin D. Sun J. Zhou G. Cui Y. (2016). Improved lithium ionic conductivity in composite polymer electrolytes with oxide-ion conducting nanowires. ACS Nano 10, 11407–11413. 10.1021/acsnano.6b06797 28024352

[B49] Liu W. Liu N. Sun J. Hsu P. C. Li Y. Z. Lee H. W.. (2015). Ionic conductivity enhancement of polymer electrolytes with ceramic nanowire fillers. Nano Lett., 15, 2740–2745. 10.1021/acs.nanolett.5b00600 25782069

[B50] Manthiram A. Yu X. W. Wang S. F. (2017). Lithium battery chemistries enabled by solid-state electrolytes. Nat. Rev. Mater. 2:16103 10.1038/natrevmats.2016.103

[B51] Meyer W. H. (1998). Polymer electrolytes for lithium-ion batteries. Adv. Mater. 10, 439-+. 10.1002/(sici)1521-4095(199804)10:6<439::Aid-adma439>3.0.Co;2-i 21647973

[B52] Mueller U. Schubert M. Teich F. Puetter H. Schierle-Arndt K. Pastre J. (2006). Metal-organic frameworks–prospective industrial applications. J. Mater. Chem. 16, 626–636. 10.1039/b511962f

[B53] Nair J. R. Gerbaldi C. Chiappone A. Zeno E. Bongiovanni R. Bodoardo S. (2009). UV-cured polymer electrolyte membranes for Li-cells: Improved mechanical properties by a novel cellulose reinforcement. Electrochem. Commun. 11, 1796–1798. 10.1016/j.elecom.2009.07.021

[B54] Nan C. W. Fan L. Z. Lin Y. H. Cai Q. (2003). Enhanced ionic conductivity of polymer electrolytes containing nanocomposite SiO_2_ particles. Phys. Rev. Lett. 91:4. 10.1103/PhysRevLett.91.266104 14754072

[B55] O'Callaghan M. P. Powell A. S. Titman J. J. Chen G. Z. Cussen E. J. (2008). Switching on fast lithium ion conductivity in garnets: The structure and transport properties of Li(3+x)Nd(3)Te(2-x)Sb(x)O(12). Chem. Mater. 20, 2360–2369. 10.1021/cm703677q

[B56] Osada I. de Vries H. Scrosati B. Passerini S. (2016). Ionic-liquid-based polymer electrolytes for battery applications. Ange. Chem. Int. Ed. 55, 500–513. 10.1002/anie.201504971 26783056

[B57] Pal P. Ghosh A. (2018). Influence of TiO2 nano-particles on charge carrier transport and cell performance of PMMA-LiClO_4_ based nano-composite electrolytes. Electrochim. Acta 260, 157–167. 10.1016/j.electacta.2017.11.070

[B58] Perez-Estebanez M. Isasi-Marin J. Toebbens D. M. Rivera-Calzada A. Leon C. (2014). A systematic study of Nasicon-type Li-i + XMXTi2 _ x(PO4)(3) (M: Cr, Al, Fe) by neutron diffraction and impedance spectroscopy. Solid State Ionics 266, 1–8. 10.1016/j.ssi.2014.07.018

[B59] Qiu W.-L. Yang Q.-H. Ma X.-h. Fu Y.-B. Zong X.-F. (2004). Research on PEO-based dry solid polymer electrolytes for rechargeable lithium batteries. Chin. J. Power Sources 28, 440–448, 457.

[B60] Quartarone E. Mustarelli P. (2011). Electrolytes for solid-state lithium rechargeable batteries: recent advances and perspectives. Chem. Soc. Rev. 40, 2525–2540. 10.1039/c0cs00081g 21253642

[B61] Ratner M. A. Johansson P. Shriver D. F. (2000). Polymer electrolytes: ionic transport mechanisms and relaxation coupling. Mrs Bullet. 25, 31–37. 10.1557/mrs2000.16

[B62] Reddy M. J. Chu P. P. Kumar J. S. Rao U. V. S. (2006). Inhibited crystallization and its effect on conductivity in a nano-sized Fe oxide composite PEO solid electrolyte. J. Power Sources, 161, 535–540. 10.1016/j.jpowsour.2006.02.104

[B63] Scrosati B. Garche J. (2010). Lithium batteries: status, prospects and future. J. Power Sources, 195, 2419–2430. 10.1016/j.jpowsour.2009.11.048

[B64] Sheng J. Tong S. He Z. Yang R. (2017). Recent developments of cellulose materials for lithium-ion battery separators. Cellulose 24, 4103–4122. 10.1007/s10570-017-1421-8

[B65] Sheng O. Jin C. Luo J. Yuan H. Huang H. Gan Y.. (2018). Mg_2_B_2_O_5_ Nanowire enabled multifunctional solid-state electrolytes with high ionic conductivity, excellent mechanical properties, and flame-retardant performance. Nano Lett. 18, 3104–3112. 10.1021/acs.nanolett.8b00659 29692176

[B66] Shi Q. X. Xia Q. Xiang X. Ye Y. S. Hai Yan P. Xue Z. G.. (2017). Self-assembled polymeric ionic liquid-functionalized cellulose nano-crystals: constructing 3D ion-conducting channels within ionic liquid-based composite polymer electrolytes. Chem. Euro. J. 23, 11881–11890. 10.1002/chem.201702079 28613388

[B67] Siqueira L. J. A. Ribeiro M. C. C. (2006). Molecular dynamics simulation of the polymer electrolyte poly(ethylene oxide)/LiClO4. II. Dynamical properties. J. Chem. Phys. 125:214903 10.1063/1.2400221 17166045

[B68] Srivastava N. Tiwari T. (2009). New trends in polymer electrolytes: a review. E-Polymers 146, 1–17. 10.1515/epoly.2009.9.1.1738

[B69] Stavila V. Talin A. A. Allendorf M. D. (2014). MOF-based electronic and optoelectronic devices. Chem. Soc. Rev. 43, 5994–6010. 10.1039/c4cs00096j 24802763

[B70] Stephan A. M. Nahm K. S. (2006). Review on composite polymer electrolytes for lithium batteries. Polymer 47, 5952–5964. 10.1016/j.polymer.2006.05.069

[B71] Stramare S. Thangadurai V. Weppner W. (2003). Lithium lanthanum titanates: A review. Chem. Mater. 15, 3974–3990. 10.1021/cm0300516

[B72] Subianto S. Mistry M. K. Choudhury N. R. Dutta N. K. Knout R. (2009). Composite polymer electrolyte containing ionic liquid and functionalized polyhedral oligomeric silsesquioxanes for anhydrous PEM applications. ACS Appl. Mater. Interfaces 1, 1173–1182. 10.1021/am900020w 20355910

[B73] Sun B. Mindemark J. Edstrom K. Brandell D. (2014). Polycarbonate-based solid polymer electrolytes for Li-ion batteries. Solid State Ionics 262, 738–742. 10.1016/j.ssi.2013.08.014

[B74] Sun J. Z. MacFarlane D. R. Forsyth M. (1996). Ion conductive poly(ethylene oxide dimethyl siloxane) copolymers. J. Polymer Sci. Polymer Chem. 34, 3465–3470.

[B75] Tambelli C. C. Bloise A. C. Rosario A. Pereira E. C. Magon C. J. Donoso J. P. (2002). Characterisation of PEO-Al2O3 composite polymer electrolytes. Electrochim. Acta 47, 1677–1682. 10.1016/s0013-4686(01)00900-8

[B76] Tang Z. Hu L. Zhang Z. Su F. (2007). Research progress of solid polymer electrolytes for lithium ion batteries. J. Chin. Ceramic Soc. 35, 123–128.

[B77] Thangadurai V. Kaack H. Weppner W. J. F. (2003). Novel fast lithium ion conduction in garnet-type Li_5_La_3_M_2_O_12_ (M = Nb, Ta). J. Am. Ceramic Soc. 86, 437–440. 10.1111/j.1151-2916.2003.tb03318.x

[B78] Thokchom J. S. Gupta N. Kumar B. (2008). Superionic conductivity in a lithium aluminum germanium phosphate glass-ceramic. J. Electrochem. Soc. 155, A915–A920. 10.1149/1.2988731

[B79] Tikekar M. D. Choudhury S. Tu Z. Archer L. A. (2016). Design principles for electrolytes and interfaces for stable lithium-metal batteries. Nat. Energy 1, 1–7. 10.1038/nenergy.2016.114

[B80] Verma P. Maire P. Novak P. (2010). A review of the features and analyses of the solid electrolyte interphase in Li-ion batteries. Electrochim. Acta 55, 6332–6341. 10.1016/j.electacta.2010.05.072

[B81] Villaluenga I. Wujcik K. H. Tong W. Devaux D. Wong D. H. C. DeSimone J. M.. (2016). Compliant glass-polymer hybrid single ion-conducting electrolytes for lithium batteries. Proc. Natl. Acad. Sci. USA. 113, 52–57. 10.1073/pnas.1520394112 26699512 PMC4711862

[B82] Wang L.-P. Zhang X.-D. Wang T.-S. Yin Y.-X. Shi J.-L. Wang C.-R. (2018). Ameliorating the interfacial problems of cathode and solid-state electrolytes by interface modification of functional polymers. Adv. Energy Mater. 8:1801528 10.1002/aenm.201801528

[B83] Wang X. Zhang M. Alvarado J. Wang S. Sina M. Lu B.. (2017). new insights on the structure of electrochemically deposited lithium metal and its solid electrolyte interphases via cryogenic TEM. Nano Lett. 17, 7606–7612. 10.1021/acs.nanolett.7b03606 29090936

[B84] Wang Z. Wang S. Wang A. Liu X. Chen J. Zeng Q. (2018). Covalently linked metal-organic framework (MOF)-polymer all-solid-state electrolyte membranes for room temperature high performance lithium batteries. J. Mater. Chem. A 6, 17227–17234. 10.1039/c8ta05642k

[B85] Watanabe M. Endo T. Nishimoto A. Miura K. Yanagida M. (1999). High ionic conductivity and electrode interface properties of polymer electrolytes based on high molecular weight branched polyether. J. Power Sources 81, 786–789. 10.1016/s0378-7753(99)00250-5

[B86] Wei-Min W. (2012). Study on all solid-state composite polymer electrolyte. Adv. Mat. Res. 571, 13–16. 10.4028/www.scientific.net/AMR.571.13

[B87] Weston J. E. Steele B. C. H. (1982). Effects of inert fillers on the mechanical and electrochemical properties of lithium salt poly (ethylene-oxide) polymer electrolytes. Solid State Ionics 7, 75–79. 10.1016/0167-2738(82)90072-8

[B88] Wu J.-F. Pang W. K. Peterson V. K. Wei L. Guo X. (2017). Garnet-type fast Li-ion conductors with high ionic conductivities for all-solid-state batteries. ACS Appl. Mater. Interfaces 9, 12461–12468. 10.1021/acsami.7b00614 28332828

[B89] Xie H. Yang C. Fu K. Yao Y. Jiang F. Hitz E. (2018). Flexible, scalable, and highly conductive garnet-polymer solid electrolyte templated by bacterial cellulose. Adv. Energy Mater. 8:1703474 10.1002/aenm.201703474

[B90] Xie X.-C. Huang K.-J. Wu X. (2018). Metal-organic framework derived hollow materials for electrochemical energy storage. J. Mater. Chem. A 6, 6754–6771. 10.1039/c8ta00612a

[B91] Xiong H. M. Wang Z. D. Xie D. P. Cheng L. Xia Y. Y. (2006). Stable polymer electrolytes based on polyether-grafted ZnO nanoparticles for all-solid-state lithium batteries. J. Mater. Chem. 16, 1345–1349. 10.1039/b514346b

[B92] Xu K. (2004). Nonaqueous liquid electrolytes for lithium-based rechargeable batteries. Chem. Rev. 104, 4303–4417. 10.1021/cr030203g 15669157

[B93] Xu R. C. Xia X. H. Zhang S. Z. Xie D. Wang X. L. Tu J. P. (2018). Interfacial challenges and progress for inorganic all-solid-state lithium batteries. Electrochim. Acta 284, 177–187. 10.1016/j.electacta.2018.07.191

[B94] Yang L. Wang Z. Feng Y. Tan R. Zuo Y. Gao R. (2017). Flexible composite solid electrolyte facilitating highly stable soft contacting Li-electrolyte interface for solid state lithium-ion batteries. Adv. Energy Mater. 7:1701437 10.1002/aenm.201701437

[B95] Yarmolenko O. V. Yudina A. V. Khatmullina K. G. (2018). Nanocomposite polymer electrolytes for the lithium power sources (a Review). Rus. J. Electrochem. 54, 325–343. 10.1134/s1023193518040092

[B96] Young W. S. Kuan W. F. Epps T. H. (2014). Block copolymer electrolytes for rechargeable lithium batteries. J. Polymer Sci. B Polymer Phys. 52, 1–16. 10.1002/polb.23404

[B97] Yuan C. Li J. Han P. Lai Y. Zhang Z. Liu J. (2013). Enhanced electrochemical performance of poly(ethylene oxide) based composite polymer electrolyte by incorporation of nano-sized metal-organic framework. J. Power Sources 240, 653–658. 10.1016/j.jpowsour.2013.05.030

[B98] Zachman M. J. Tu Z. Choudhury S. Archer L. A. Kourkoutis L. F. (2018). Cryo-STEM mapping of solid-liquid interfaces and dendrites in lithium-metal batteries. Nature 560, 345–349. 10.1038/s41586-018-0397-3 30111789

[B99] Zhang J. Yue L. Hu P. Liu Z. Qin B. Zhang B.. (2014). Taichi-inspired rigid-flexible coupling cellulose-supported solid polymer electrolyte for high-performance lithium batteries. Sci. Rep. 4:6272. 10.1038/srep06272 25183416 PMC4152750

[B100] Zhang Q. Q. Liu K. Ding F. Liu X. J. (2017). Recent advances in solid polymer electrolytes for lithium batteries. Nano Res. 10, 4139–4174. 10.1007/s12274-017-1763-4

[B101] Zhang X. Liu T. Zhang S. Huang X. Xu B. Lin Y.. (2017). Synergistic Coupling between Li_6.75_La_3_Zr_1.75_Ta_0.25_O_12_ and Poly(vinylidene fluoride) induces high ionic conductivity, mechanical strength, and thermal stability of solid composite electrolytes. J. Am. Chem. Soc. 139, 13779–13785. 10.1021/jacs.7b06364 28898065

[B102] Zhang X.-Q. Cheng X.-B. Zhang Q. (2018). Advances in interfaces between Li metal anode and electrolyte. Adv. Mater. Interfaces 5:1701097 10.1002/admi.201701097

[B103] Zhao Y. Huang Z. Chen S. Chen B. Yang J. Zhang Q. (2016a). A promising PEO/LAGP hybrid electrolyte prepared by a simple method for all-solid-state lithium batteries. Solid State Ionics 295, 65–71. 10.1016/j.ssi.2016.07.013

[B104] Zhao Y. Wu C. Peng G. Chen X. Yao X. Bai Y. (2016b). A new solid polymer electrolyte incorporating Li_10_GeP_2_S_12_ into a polyethylene oxide matrix for all-solid-state lithium batteries. J. Power Sources 301, 47–53. 10.1016/j.jpowsour.2015.09.111

[B105] Zhao Y. Zhang Y. Gosselink D. Doan T. N. L. Sadhu M. Cheang H.-J.. (2012). Polymer electrolytes for lithium/sulfur batteries. Membranes 2, 553–564. 10.3390/membranes2030553 24958296 PMC4021916

[B106] Zhu P. Yan C. Dirican M. Zhu J. Zang J. Selvan R. K. (2018). Li_0.33_La_0.557_TiO_3_ ceramic nanofiber-enhanced polyethylene oxide-based composite polymer electrolytes for all-solid-state lithium batteries. J. Mater. Chem. A 6, 4279–4285. 10.1039/c7ta10517g

